# Supplementary material for: Validation of a UPLC-MS/MS Method for Quantifying Intracellular Olaparib Levels in Resistant Ovarian Cancer Cells
Source: Pharmaceuticals (Basel). 2025 Dec 8;18(12):1870. doi: 10.3390/ph18121870 (PMC12736094; doi:10.3390/ph18121870)
Supplement: Supplementary file 1 [file pharmaceuticals-18-01870-s001.zip › pharmaceuticals-3976458-supplementary.pdf]

Supplementary material

Article

Validation of a UPLC-MS/MS Method for Quantifying Intracellular Olaparib Levels in Resistant Ovarian Cancer Cells

Szymon W. Kmiecik [1], Jennifer Lewis [2,3], Jonas Schwickert [2,3], Henrik Breitenreicher [1], Martin R. Sprick [2,3] and Jürgen Burhenne [1,*]

[1] Internal Medicine IX—Department of Clinical Pharmacology and Pharmacoepidemiology, Medical Faculty Heidelberg, Heidelberg University Hospital, Heidelberg University, Im Neuenheimer Feld 410, 69120 Heidelberg, Germany
[2] Division of Stem Cells and Cancer, German Cancer Research Center (DKFZ), DKFZ-ZMBH Alliance, Im Neuenheimer Feld 280, 69120 Heidelberg, Germany
[3] Heidelberg Institute for Stem Cell Technology and Experimental Medicine (HI-STEM gGmbH), Im Neuenheimer Feld 280, 69120 Heidelberg, Germany
* Correspondence: juergen.burhenne@med.uni-heidelberg.de; Tel.: +49-6221-56-36395; Fax: +49-6221-56-5832

Table S1. Calibration curves of validation batches calculated with linear regression and $1/x^2$ weighting.

Validation batch	r^2	Equations
#1	0.9975	y = 1.13996*x + 0.00073
#2	0.9963	y = 1.15938*x + 0.03547
#3	0.9966	y = 1.11903*x + 0.01832

Table S2. SIL-IS normalized matrix effect and recovery data of the cell validation.

QC level	Nominal concentration [ng/mL]	Matrix effect		Recovery	
		p388	OC12	p388	OC12
QC-A	3	100.2%	106.2%	110.4%	104.1%
QC-C	225	104.1%	103.8%	102.4%	102.7%

QC: quality control, n ≥ 4 replicates

Table S3. SIL-IS normalized dilution integrity analysis.

QC level	Nominal concentration [ng/mL]	Dilution factor	Dilution integrity
QC-C	225	5	106.6%

Ratio between diluted and undiluted QC-C is shown, QC: quality control, n = 6 replicates

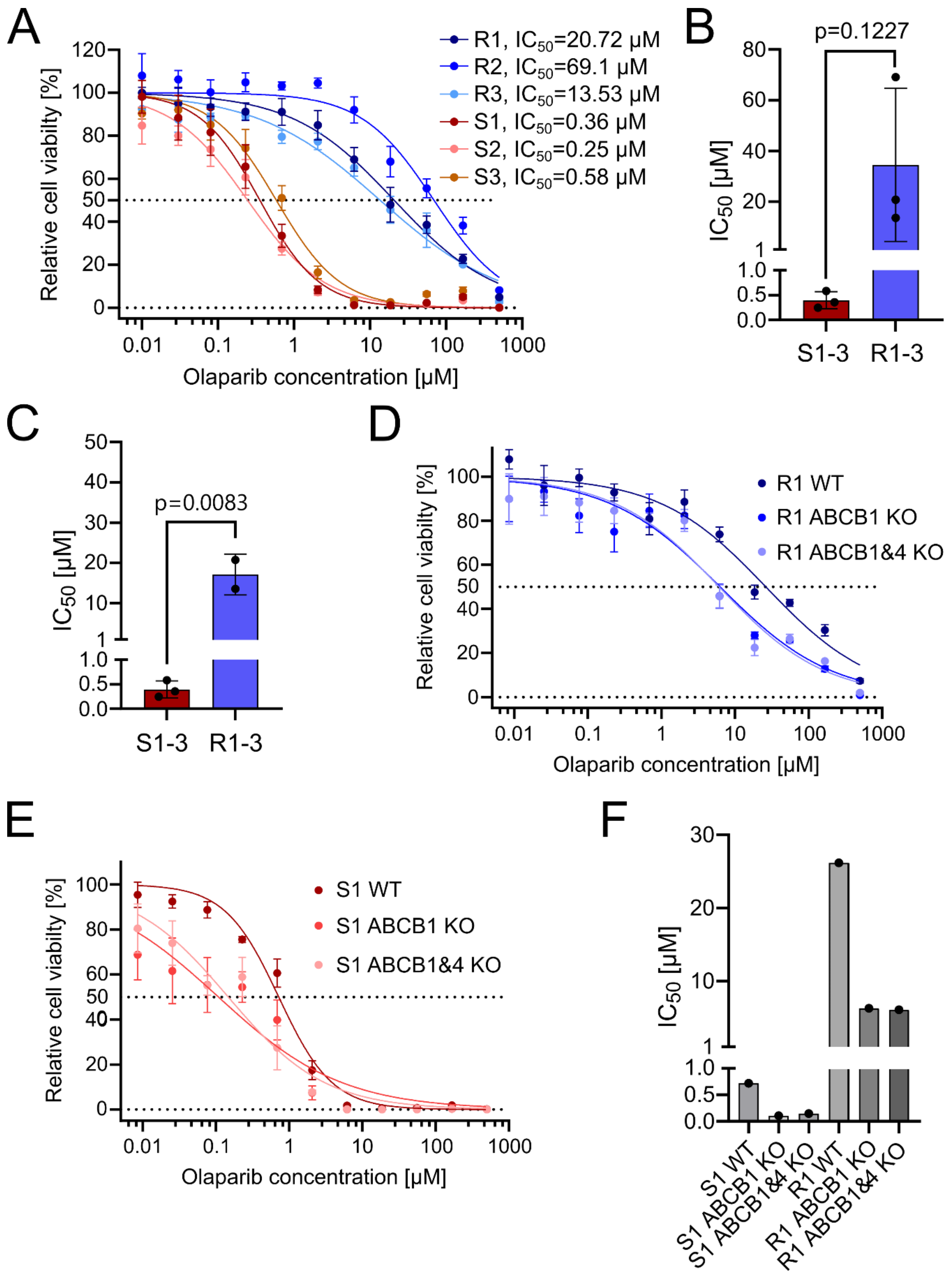

Supplementary Figure S1 (related to Figure 1 and Figure 2). OC12 cells with acquired resistance to olaparib; KO of ABCB1/4 could only slightly reduce the PARPi resistance.

A, Cell viability assay results for olaparib sensitive (S1-3) and resistant (R1-3) OC12 cell lines after the 10th treatment round. Cells were treated for 5 days with specified concentrations of olaparib, and relative cell viability was normalized to the corresponding DMSO control, mean value ± SD is shown, n = 4 replicates, data were fitted in GraphPad Prism with normalized response variable slope model. IC_{50} value for each cell line is indicated in the figure;

B, IC_{50} values for OC12 sensitive (S1-3) and resistant (R1-3) cell lines after 10 olaparib treatment rounds, n = 3, mean value ± SD is shown, unpaired T-test was used to evaluate statistical significance (p value = 0.1227).

C, IC_{50} values from S1A for OC12 sensitive (S1-3) and resistant (R1&3) cell lines after 10 olaparib treatment rounds, n ≥ 2, mean value ± SD is shown, unpaired T-test was used to evaluate statistical significance (p value = 0.0083);

D, Cell viability assay of OC12 R1 cell line (WT, ABCB1 KO, and ABCB1&4 KO). Cells were treated for 5 days with specified concentrations of olaparib, and relative cell viability was normalized to the corresponding DMSO control, mean value ± SD is shown, n ≥ 3, data were fitted in GraphPad Prism with normalized response variable slope model;

E, Cell viability assay of OC12 S1 cell line (WT, ABCB1 KO, and ABCB1&4 KO). Cells were treated for 5 days with indicated concentrations of olaparib, and relative cell viability was normalized to the corresponding DMSO control, mean value ± SD is shown, n ≥ 3, data were fitted in GraphPad Prism with normalized response variable slope model;

F, IC_{50} values calculated from S1C and S1D for OC12 sensitive (S1) and resistant (R1) cell lines (WT, ABCB1 KO, and ABCB1&4 KO) treated with olaparib, n = 1, mean value is shown.

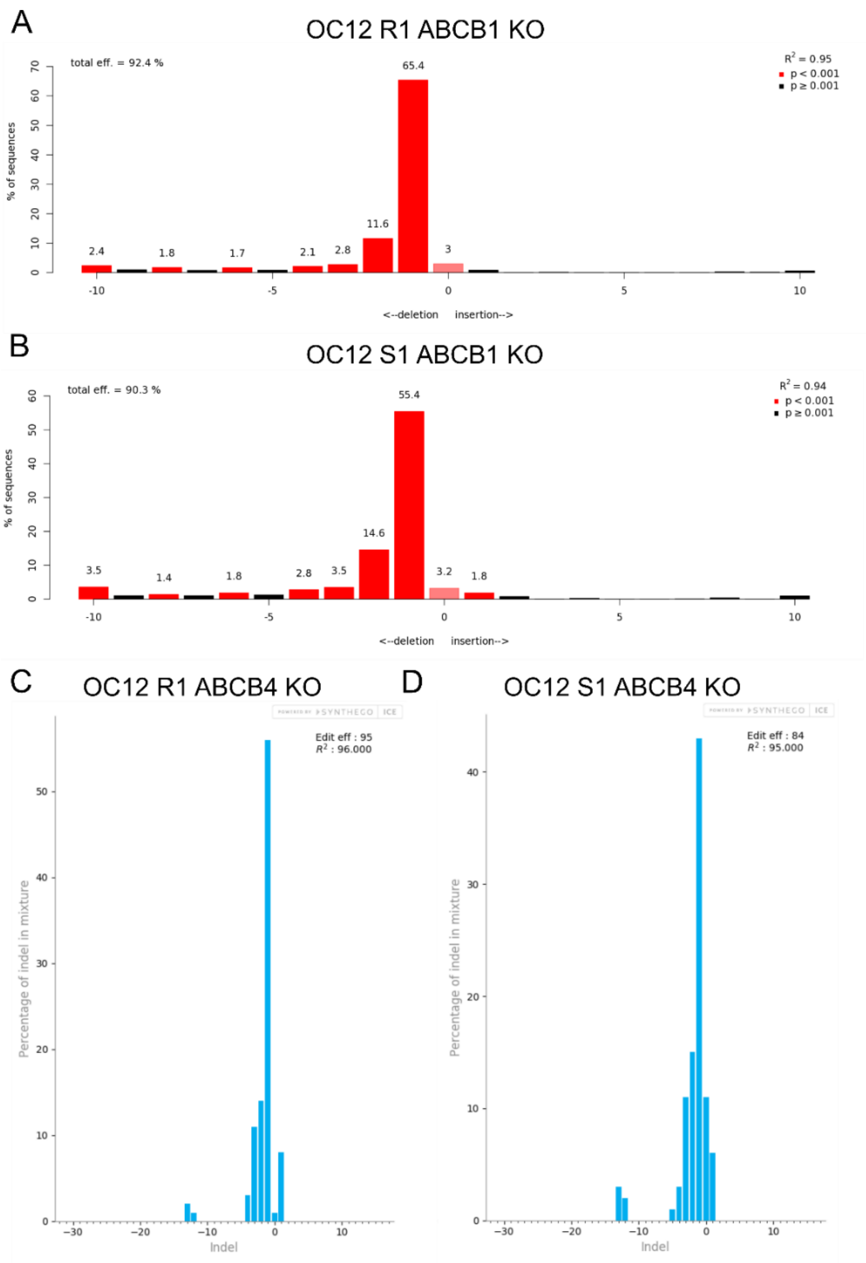

Supplementary Figure S2 (related to Figure 2 and Figure S1). Validation of ABCB1 and ABCB4 CRISPR-Cas9 knockouts using Sanger sequencing.

Reading frame shift following a knockout is reflected by the proportion of sequences containing out-of-frame insertions or deletions detected by Sanger sequencing.

A, ABCB1 KO efficiency in OC12 R1 cells, based on Sanger sequencing;

B, ABCB1 KO efficiency in OC12 S1 cells, based on Sanger sequencing;

C, ABCB4 KO efficiency in OC12 R1 cells, based on Sanger sequencing;

D, ABCB4 KO efficiency in OC12 S1 cells, based on Sanger sequencing.

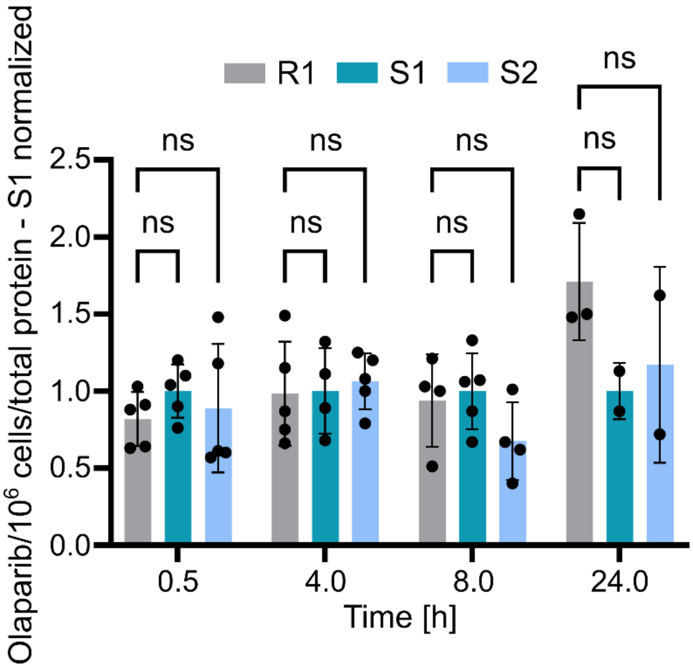

Supplementary Figure S3 (related to Figure 5). Olaparib detection in OC12 sensitive and resistant cells.

Amount of olaparib in OC12 cells measured in ng per 10^6 cells, normalized to total protein concentration and then, in addition, to olaparib sensitive (S1) samples. Normalization was done within each timepoint. Single datapoints represent technical replicates. Mean value ± SD is shown, n ≥ 4 for timepoints 0.5, 4, and 8 hours, n ≥ 2 for 24-hour timepoint, 2-way ANOVA was used for statistical analysis, ns: not significant. Protein concentration was monitored for 2 independent experiments. The 24-hour timepoint was analyzed in only one experiment.

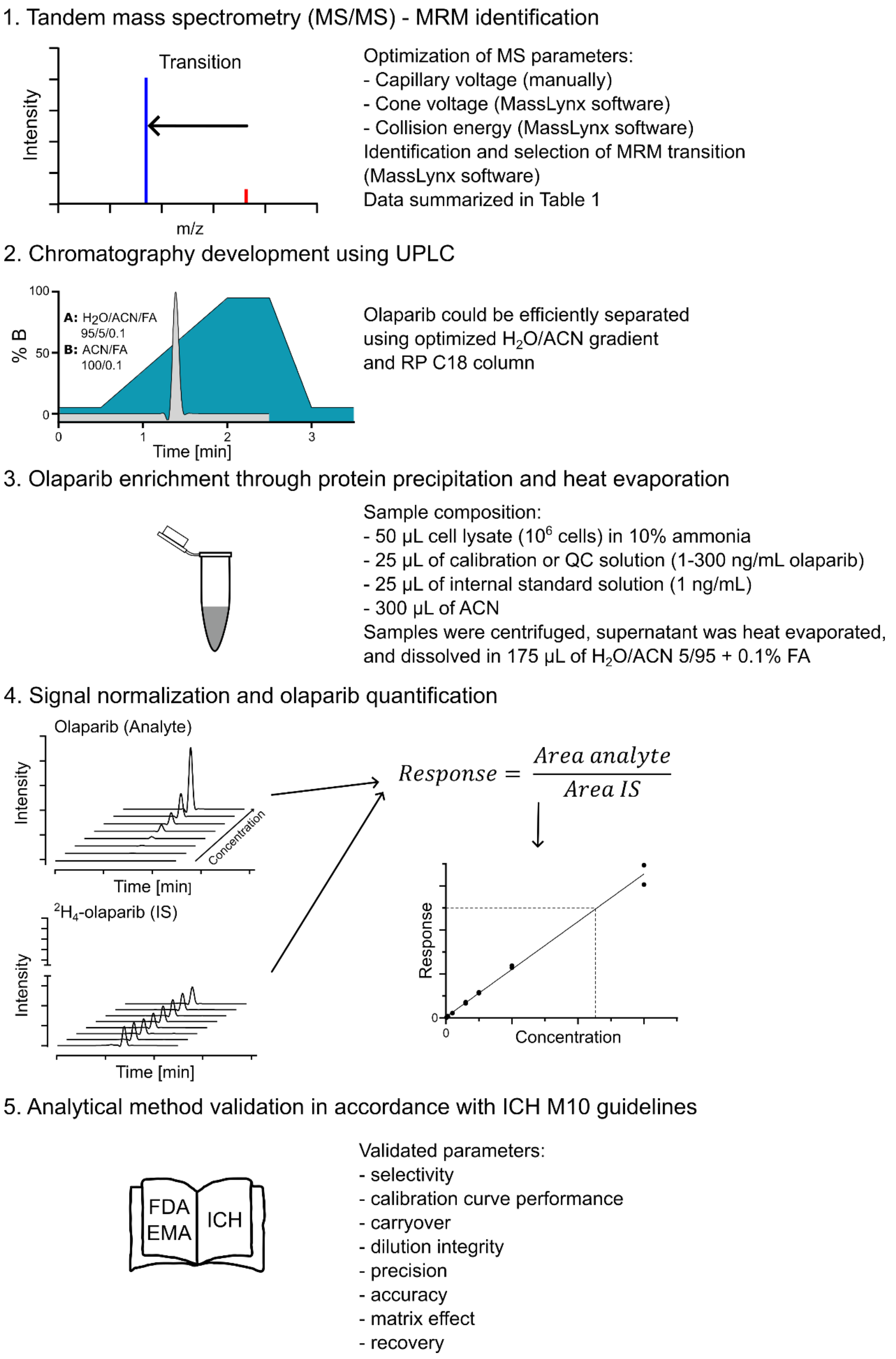

Supplementary Figure S4 (related to Material and methods). Outline of analytical method development and validation for olaparib.

1, In the initial step, mass spectrometry parameters for olaparib detection were optimized. The obtained results are summarized in Table 1;

2, To achieve efficient separation from potential interfering molecules, reversed-phase (RP) chromatography using a C18 column was applied;

3, Olaparib samples were subjected to protein precipitation with acetonitrile (ACN) to deplete proteins. Samples were then concentrated through heat evaporation and reconstitution in a high water-content solvent suitable for RP chromatography;

4, To quantify the olaparib content in the samples, the signal for olaparib (calculated as the area under the chromatogram peak) was divided by the constant signal of the olaparib internal standard (IS) in each sample, thereby calculating the analytical platform's response to olaparib at every calibration level. The resulting calibration curve was used to determine olaparib concentration in the analyzed samples;

5, The method was validated in accordance with ICH M10 guidelines for bioanalytical method validation.
